# Supplementary material for: In silico immune infiltration profiling combined with functional enrichment analysis reveals a potential role for naïve B cells as a trigger for severe immune responses in the lungs of COVID-19 patients
Source: PLoS One. 2020 Dec 2;15(12):e0242900. doi: 10.1371/journal.pone.0242900 (PMC7710067; doi:10.1371/journal.pone.0242900)
Supplement: S1 Data — (PDF) [file pone.0242900.s001.pdf]

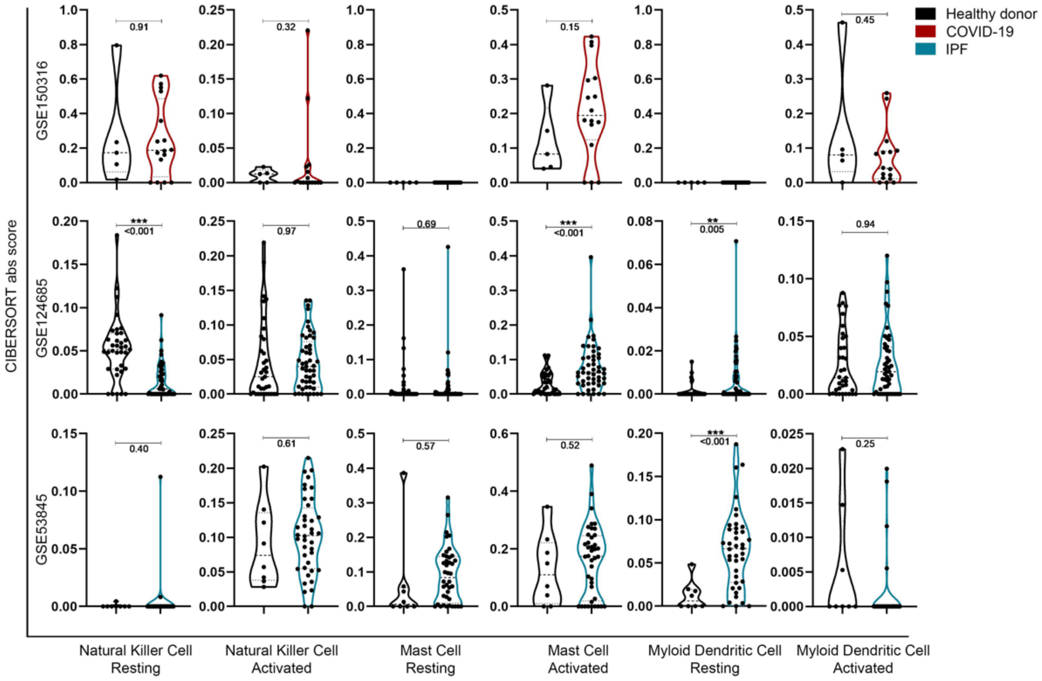

Supplementary Figure 1 In silico simulated uncategorized immune cell infiltration in lung tissue between COVID-19 and IPF patients. **P*<0.05, ***P*<0.01 and ****P*<0.001, with comparisons indicated by brackets

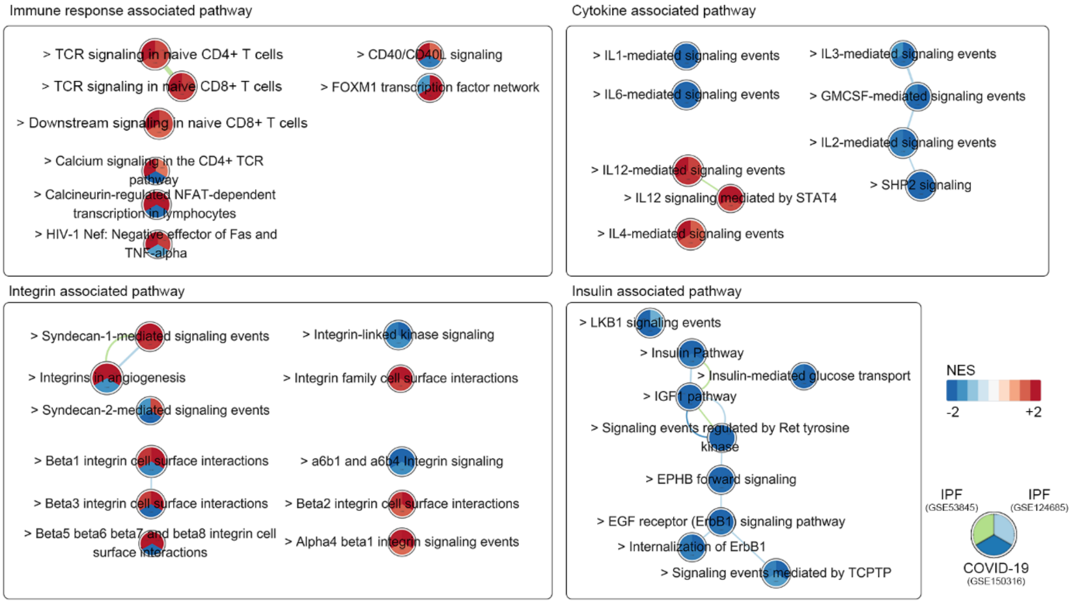

Supplementary Figure 2 PID pathway functional enrichment map summary of GSEA in the lung tissues of COVID-19 and IPF patients. Commonality of positive or negative enrichments in the PID pathway gene sets after GSEA assessment using enrichment map visualization in COVID-19 and IPF. Color represents the NES of the gene set in the database as assessed by GSEA. Gene sets that are associated with immune responses or that have a high degree of consistency and overlap in enrichment

are listed here. Criteria for enrichment significance screening: *P*-value <0.05, FDR <0.25

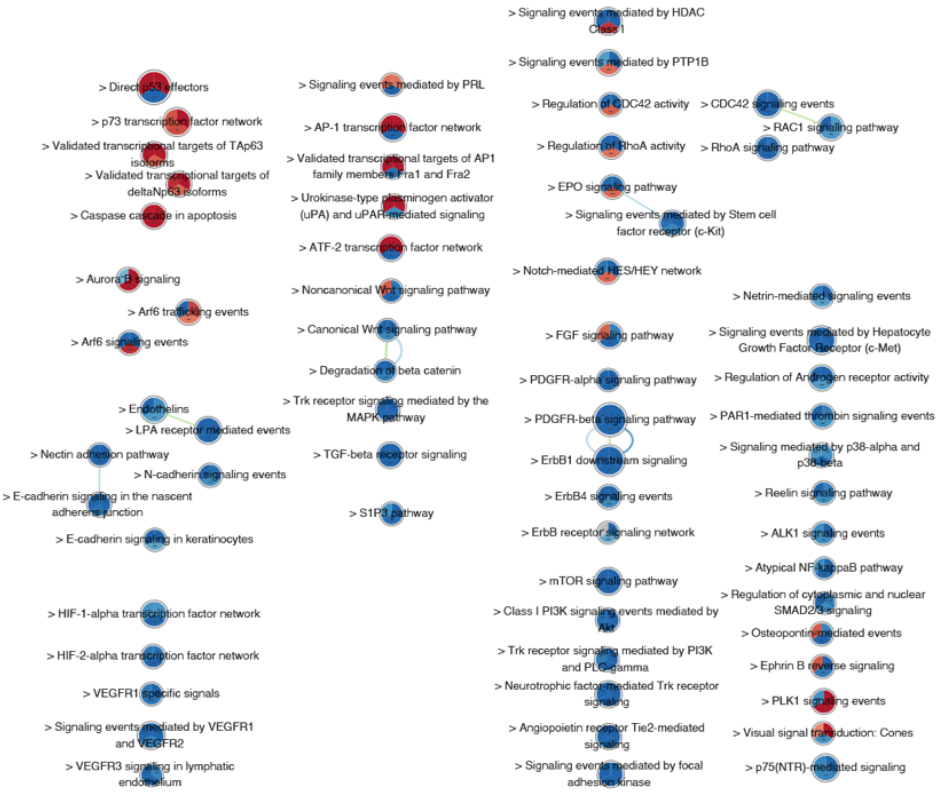

Supplementary Figure 3 Uncategorized PID pathway functional enrichment map summary of GSEA in the lung tissues of COVID-19 and IPF patients.
