# Supplementary material for: In silico immune infiltration profiling combined with functional enrichment analysis reveals a potential role for naïve B cells as a trigger for severe immune responses in the lungs of COVID-19 patients
Source: PLoS One. 2020 Dec 2;15(12):e0242900. doi: 10.1371/journal.pone.0242900 (PMC7710067; doi:10.1371/journal.pone.0242900)
Supplement: Attachment — Submitted filename: Rebuttal letter v1.docx [file pone.0242900.s002.docx]

**Reviewer #1: Wu et al. employed CIBERSORT method to investigate common and differences in the level of immune cell infiltration in lung tissues of COVID-19 patients compared with patients of Idiopathic Pulmonary Fibrosis (IPF). They determined that several immune cell sub-types, particularly naive B-cells, are highly infiltrated in COVID-19 patients. Besides that they reported several other important findings using CIBERSORT and gene set enrichment based analysis (Figs 2-6). Overall this is an interesting study. However, the study is suffering from lack of validation results. Authors have stated this limitation and addressed this major issue only by indicating that in the midst of COVID-19 pandemic, they are eager to provide this data that can be used for interpretation or to increase confidence in COVID-19 treatment. Although rapid data generation is a critical step during this pandemic situation; however, ideally we should not compromise with reproducibility, scientific rigor, and quality of the data.

Major comments**
=============
**1. It is understandable that the experimental validation of the major prediction results reported in this manuscript is a time consuming task; however, some levels of validation are required to determine the quality of the reported prediction results. Authors are suggested to evaluate single cell RNA-seq data and investigate whether the prediction results they obtained from the bulk RNA-seq data using the tool CIBERSORT are reproducible in the actual single cell resolution. A quick pubmed search shows, recently Liao et al. (Nature Medicine, May 2020; https://doi.org/10.1038/s41591-020-0901-9) published single cell landscape of bronchoalveolar immune cells extracted from moderate to sever patients with COVID-19 and healthy control and the data are publicly available (GSE128033 and GSM3660650). Authors are suggested to do a comprehensive literature search and check if this study or any additional studies could be used as the validation data. Furthermore, multiple single cell RNA-seq data on IPF (GSE94555 and GSE86618) are also available in the public repositories. Therefore, authors are suggested to take this opportunity and investigate whether their reported comparative results are reproducible in a completely independent validation data set.**

**2. If the single cell data are not suitable for the analysis because of some valid reasons, authors are suggested to at least evaluate independent RNA-seq data and examine whether the prediction results obtained from the training data are consistent in the independent validation data.**

**3. For the Gene set enrichment analysis FDR cut-off 0.25 was chosen to determine significantly enriched gene sets. Although this cut-off is not uncommon; however, a general trend is to use FDR < 0.05 to determine significance. I am wondering how much the results would be changed with the FDR < 0.05 compared with the current results. This is important to evaluate, particularly in the absence of validation data, because such a low cut-off value may introduce high-false positives.**

**Response:**

Dear reviewer, thank you very much for your valuable comments and suggestions on the direction of this paper, which have greatly help us improving the strength of evidence of our conclusions. Based on the suggestion, we attempted to search multiple public repositories and found the single-cell sequencing databases currently associated with COVID-19 lung-related immune cell infiltration provided by Chua et al. and Liao et al. (GSE145926), and the single-cell sequencing database associated with IPF lung immune cell infiltration published by Morse et al. (GSE128033). The GSE94555 and GSE86618 databases were also once included in our evaluation but were not used due to the small sample size. On the other hand, the GSE135893 IPF database contains a large number of single-cell analysis results, but due to the processing capacity of the equipment, it was not possible to analyze the relevant results of the B-cell population.

In order to achieve consistency with the CIBERSORT analysis to verify whether similar results occur in both the bulk RNA-seq and single-cell RNA-seq, and to allow for re-running the gene set clustering and visualization analysis with FDR < 0.05, we performed gene cluster overlap visualization analysis by matching the immune cell signature files used in CIBERSORT and converting them to Naïve or Memory specific gene sets. In addition, in order to reduce the complexity and improve the interpretation of the results, we polarized the enrichment results as red and green for COVID-19 or IPF respectively. The results of the single cell sequencing database and clinical situation analysis were added to Figure 6 and 7, while the PID-related analysis was included as supplementary data. For B-cell related signature analysis, since the number of gene sets has been filtered down to nearly 500, the use of FDR<0.05 may result in too few results, so please forgive us for adopting the more relaxed FDR<0.25 criterion in Figure 7A. The relevant amendments and new result paragraphs are described below:

**GO:BP analysis based on GSEA reveals specific enrichment of B-cell-mediated innate humoral responses in the lungs of COVID-19 patients**

To understand the similarities and differences in the functional enrichment of gene sets in the lung tissues of COVID-19 and IPF patients compared to controls, the GO:BP (Gene Ontology Biological Processing) gene set was employed to evaluate a variety of biological responses, including immune responses.

Based on the GSEA results, Figure 5A lists the functional gene sets with NES scores greater than 2 (COVID-19: Red, IPF: Green; pre-screening criteria: nom. *P* < 0.05, FDR < 0.05). The purple text shows the set of genes that are co-enriched in COVID-19 or IPF patients, including Humoral immune response mediated by circulating immunoglobulin, B cell mediated immunity, complement activation, phagocytosis recognition, positive regulation of B cell activation and B cell receptor signalign pathway ranked first. Showing that an immune response caused by B-cell mediators may be a consistent phenomenon that causes damage to lung tissue in patients with COVID-19 or IPF. Green and red text represent the separately enriched gene sets in IPF or COVID-19 respectively. A number of gene sets related to lung fibrosis, including extracellular structure organization and collagen associated processes, were enriched in IPF. At the same time, a variety of genes related to B-cell proliferation, differentiation and maturation as well as adaptive immune response were also solely associated with IPF. Interestingly, none of these gene sets associated with B-cell maturation and differentiation were prominent in COVID-19, and instead were enriched for the innate immune response and FC receptor signaling pathway. This phenomenon is consistent with the results of the CIBERSORT-X analysis.

To understand which genes affect the CIBERSORT-X algorithm more, we ranked the top 100 genes enriched in naive B cell or memory B cell within LM22 signature document and performed Venn diagram analysis (Figure 5B). There are 20 genes that are not involved in intersection in both types of B cell, we define them separately as “Naïve B cell specific signature” and “Memory B cell specific signature”. In addition, to systematically visualize the enriched gene set clusters, we performed the enrichment map app in Cytoscape and set the enriched gene cluster in COVID-19 as red and IPF as green. The visualized results showed that clusters belong to immune cell activation were divergent, while the other clusters were clearly polarized. For example, the chromatin centromere and sister segregation gene sets are enriched only in COVID-19, while others are enriched only in IPF. We further overlap the defined B cell specific signature with the visualized clusters, the graphical representation showed that memory B cell specific signature was mostly associated with multiple immune-related gene sets of IPF. In contrast, the naive B cell specific signature was associated with COVID-19 enriched immune gene sets. These results are consistent with the data from the CIBERSORT-X analysis of B cell lineage (Figure 3). That is, in the lungs of COVID-19 patients, undifferentiated naïve B cells may be the main immune cells that elicit humoral immune response, which has been observed in clinical practice[1, 2].

**Figure 5 GO:BP functional enrichment map summary of GSEA in the lung tissues of COVID-19 and IPF patients.**
(A) The top NES-ranked enrichment results of biological processing by gene ontology (GO) functional enrichment analysis in IPF or COVID-19 (NES > 2; FDR < 0.05). The normalized enrichment score (NES) was presented in the X-axis, and the names of enriched functional gene sets were shown in the Y-axis. The color gradation on the right side indicates false discovery rate (FDR). Size represents the number of genes in the gene set. Color of the text represents whether the gene set was enriched in both (purple) or in individual type of disease (COVID-19: Red, IPF: Green). (B) Venn diagram: two circles represent the intersection status of the top 100 genes from the Naive B cell (Red) and Memory B cell (Green) subtypes extracted from the LM22 immune cell gene signatures, respectively. The detailed list of genes in the intersection is shown in the table below. Enrichment map: Commonality of positive correlations in the GO:BP gene sets after GSEA assessment using enrichment map visualization in COVID-19 (GSE150316, Red) and IPF (GSE124685 and GSE53845, Green). Single color: the node is positively correlated in only one disease condition; Mixed: the node is positively correlated in two or three databases. Diamond: Naïve or Memory B cell specific signature, genes in the signature were shown in the table below. Lines connecting nodes or diamonds represent the degree of overlapping between two gene sets. Criteria for significance screening: *P*-value <0.05, FDR <0.05.

**Differences in B-cell clustering subtypes between COVID-19 and IPF patients verified in single-cell RNA sequencing databases**

CIBERSORT and similar deconvolution methods can be used to analyze the distribution of immune cells in the Bulk RNA-seq data, but the analysis of the signal pathways may not be accurate due to the overlap of activation between different cells. In order to further validate the phenomenon observed in bulk RNA-seq, we searched several publicly available single-cell RNA-sequencing databases and restricted the selecting criteria to those sampling from lung-associated body fluids or tissues. Chua et al. published single-cell RNA sequencing analysis of respiratory fluid samples from 20 patients with COVID-19 mild to severe disease, and Liao et al. published single-cell RNA sequencing analysis of bronchoalveolar lavage fluid (BALF) from 9 patients with COVID-19 mild to severe disease (GSE145926). On the other hand, Morse et al. published single-cell RNA sequencing analysis of lung tissues from IPF patients (GSE128033). According to the annotation provided by the authors, we circled B-cell populations from patients or healthy donors and evaluated the overall percentages of expression and the average standardized expression of each gene in the B-cell specific signatures. The average standardized expression of all genes was further analyzed (Figure 6). The results showed that genes in naive B cell specific signature was significantly higher in COVID-19 patient B cells, whereas genes in memory B cell specific signature were generally increased in IPF patient B cells, echoing our observations in bulk RNA-seq databases.

**Figure 6 Validation of the phenotypes of B-cell populations in single-cell RNA sequencing of lung-associated body fluid samples from COVID-19 and IPF patients.**
UMAP shows the visualized distribution of whole cells in single-cell RNA sequencing data from two COVID-19 databases (A) and IPF database (B). the “B-cell clusters” annotated by the original authors are shown in red, the unassigned cells are shown in gray. B-cell clusters are further colored red and blue to represent those from disease or healthy donors (HC) respectively. The proportion and average expression of genes related to Naive or Memory B cells in the B-cell population are shown as dot plots. Relative gene expression is shown in color and the overall B-cell expression gene percentage is shown in dot size (%). The mean Z scores of all genes’ expression in B cell populations from HC or disease groups are shown in Violin plot. Statistical significance is analyzed using Welch’s t-test approach. *: *P* < 0.05, **: *P* < 0.01, ***: *P* < 0.001. The actual *P*-values are shown under the asterisks as well.

**B cells from patients with severe COVID-19 have genetic phenotype similar to antibody-secreting cells, and the percentage of B cell infiltration appears to correlate with the severity of the disease.**

Given the results of the bulk RNA seq analysis, we were curious whether any specific distinctions existed between the B-cell lineage populations of patients with mild or severe COVID-19. Firstly, we divided the B cells from COVID-19 in two single-cell databases by disease severity and analyzed them by GSEA together with gene sets from C7 immune signature that belonged to B cell lineage, and then we cross match the two databases with the gene set enriched in moderate disease. The results showed that B cells from COVID-19 patients with severe symptoms had a tendency to be antibody secreting cells, such as plasma cells, plasmablast, IgM memory B cells, Follicular B cells, and unstimulated B cells compared to those from patients with mild symptoms (Figure 7A). Taken together with the aforementioned observations, we hypothesized that B cells from the lungs of COVID-19 critically ill patients may be associated with DN2 B cells, which have been considered in recent years to be the main inducer of extrafollicular response [3, 4]. In-depth analysis of DN2 B-cell related genes revealed that the overall expression ratio and average expression of genes that were thought to be reduced in DN2 B cells, such as CD21, CXCR5, IRF8 and TRAF5, were lower in B cells from critically ill patients, especially in samples from BALF (Figure 7B). Further, we analyzed the ratio of B-cell infiltration and the clinical characteristics of the patients and showed that when B-cell infiltration > 1%, there was a longer hospitalization and ICU duration, a higher proportion of severe disease (66.7%), a higher proportion of no recovery (33.3%) and a higher proportion of multi-organ failure (55.6%), indicating an association between increased B-cell infiltration and severity of disease in COVID-19 patients (Figure 7C).

**Figure 7 Assessing the phenotypic and clinical status differences of B-cell populations between mild and severe symptoms of COVIF-19.**

(A) Schematic diagram of the GSEA analyzing process for B cells of mild and severe COVID-19 patients in different databases. GSEA results were cross-referenced and the overlapping enriched data sets of the two databases were listed. The NES scores of each gene set are additionally represented by shades of red to represent the relative enrichment level. Those considered relevant to the B-cell population of patients with severe COVID-19 in the two immune cell comparisons are marked in red. (B) The dot plots represent the overall expression ratio and average expression of genes thought to be related to antibody-secreting cells and DN2 B cells. The overall expression percentage is represented by the size of the dots and the average expression is represented by the color. (C) Comparison of B-cell infiltration ratio and clinical status of patients with COVID-19. Box plots are shown for patients with B cell infiltration ratio < 1% (Black) or > 1% (Red) for duration of hospitalization and ICU. Pie charts show the severity, recovery and complications of multi-organ failure in patients with different levels of B-cell infiltration.

**Reviewer #2: Summary:
This is a timely manuscript presenting a comparative in silico analysis of estimated cell type frequencies and gene expression changes in the lungs of deceased COVID-19 patients and idiopathic pulmonary fibrosis patients. Using CIBERSORT to estimate cell subset abundancies from gene expression profiles, the authors find certain cell types, such as monocytes and naïve B cells, uniquely increased in fatal COVID-19 samples. Furthermore, gene set enrichment analysis revealed changes in functional and signaling pathways, including decreases in CD40/CD40L signaling and alterations in integrin expression. The authors suggest that these results implicate naïve B cells as a potential mediator of lung pathology in COVID-19. Overall, this manuscript presents original research and the conclusions are supported by the data. However, there are some concerns about the limitations of the study and presentation of the results that should be addressed prior to acceptance.**

Major concerns:
**1. The title overstates the findings and should be changed to reflect the fact that the role for naïve B cells in COVID-19 lung pathology is not proven by this analysis. A potential title could be: “In silico immune infiltration profiling combined with functional enrichment analysis [suggests a/reveals a potential] role for naïve B cells as a trigger for severe immune responses in the lungs of COVID-19 patients”.**

**Response:**

Many thanks to the valuable suggestion, we have corrected the title as follows: “In silico immune infiltration profiling combined with functional enrichment analysis reveals a potential role for naïve B cells as a trigger for severe immune responses in the lungs of COVID-19 patients.”

**2. A significant limitation which is not addressed is that all of the COVID-19 samples analyzed were from fatal cases. To understand whether naïve B cell infiltration plays a specific role in the pathology of severe/fatal COVID-19, it would be important to examine whether these changes also occur in mild/moderate cases where there is a lack of significant lung damage or fibrosis. If the samples/data are not available to make this comparison, this limitation should be raised in the discussion.**

**Response:**

We thank the reviewer for the suggestion. For moderate/severe case comparison, we analyzed two single cell RNA-seq databases that clearly indicate the severity of COVID-19, and further compared the phenotypic differences between B cells from different conditions and their degree of infiltration in relation to clinical status in Figure 7. The relevant amendments are described below.

**3. The authors state the following rationale for comparing COVID-19 and IPF: “Nearly all of the patients who died from COVID-19 had severe lung tissue damage and pulmonary fibrosis[29]. On the other hand, mortality in IPF is generally the result of progressive fibrotic lung disease.” If pulmonary fibrosis is a cause of mortality in both cases, it would make sense to look for common signatures rather than those unique to COVID-19. I think further clarification on the rationale here would be useful.**

**Response:**

We thank the reviewer for the suggestions and strongly agree that it is necessary and valuable to analyze the shared signatures of the two diseases. However, given the availability of raw data and the complex interactions of multiple immune cells, we are currently unable to analyze such a large amount of data and further expand the presentation of the results. For example, the data tentatively suggest that differences in macrophage are common to both diseases, a phenomenon also observed in our newly added single cell sequencing database, but a complete analysis of all macrophage-related gene set signatures and correlations would require more time, knowledge, and manpower to achieve. On the other hand, we did observe some possible disease-related findings, such as the suppression of insulin-associated signaling pathways in both diseases, which we believe may be related to the abnormal regulation of blood glucose in patients with pulmonary fibrosis, but decided to delete this discussion due to space limitation and lack of expertise. As such, we are grateful for your understanding of our decision to focus on the specific differences between the two diseases. Hopefully, the publication of this paper will allow more experts in various fields to conduct in-depth analysis on different faces.

**4. Figures 2-4: CIBERSORT can return relative cellular fractions instead of absolute score. These would be easier to interpret as an estimated percentage of total cells. In some cases the y axis scale changes significantly between datasets, making it harder to compare. Additionally, as a positive control it would be good to show that the relative cellular fractions of different subsets in the healthy samples match the approximate expected distribution of cell types in normal lung tissue. This could be included as a supplementary figure.**

**Response：**

Thank you very much for your suggestion and we couldn't agree with you more. We have evaluated the relative scores using Cibersort-X, but the presentation of the results may be affected by the over-representation of specific immune cells, which significantly reduces the impact of the analysis of the target cell clusters. On the other hand, there are other recent publications that analyzed the same database using different deconvolution strategies, and we decided not to include the presentation and discussion of relative cell scores in this paper due to the issue of repetitive publication [5].

**5. Figure 5: Given that there are ~7000 GO gene sets, an FDR<0.25 cutoff is quite relaxed and will likely result in many false positives. Do the results change if using a stricter cutoff such as 0.05?**

**Response：**

Thanks to the reviewer's suggestion, we have recreated Figure 5 under stricter conditions, as described below.

**6. Figure 5-6: In panel 5A, it appears the results from the two IPF datasets have been merged, but in panel 5B and Figure 6 they are separated. The authors should merge these results as well in panel 5B/Figure 6 to simplify the visualization and make a clearer message. Furthermore, the method of merging is not described. I would suggest running the enrichment analysis separately in each dataset and identifying gene sets that are consistently enriched in both datasets to produce the most robust results.**

**Response：**

Thanks to the reviewer's suggestion, we have recreated Figure 5, which combines the IPF database results and makes the visualization easier to interpret. All corrections and results will be described below.

**7. Figure 5B: The figure legend mentions “positive correlations”. If these are correlations, what are the variables being correlated? Instead, are these gene set enrichments? If so, the authors should change “positive correlations” to “positive enrichments” or “upregulated pathways versus healthy”. The legend also states that the edge represent overlap in genes between different gene sets. If this is so, how can an edge be dataset specific (colored), since the members of a gene set are predefined and not dataset dependent?**

**Response：**

Thanks to the reviewer for the correction, this is indeed an error in our narration. On the one hand, the phenomenon of edge appearing colored no longer appears after updating the enrichment map, which may be a version-specific anomaly that causes us to misinterpret the results. The above error has been corrected after reproduction and is described below.

**8. Figures 5/6: As is suggested by the differences in estimated cell frequencies in Figures 2-4, the changes in gene expression between COVID-19/IPF and healthy can be driven both by alterations in cell type abundancies and by transcriptional changes within a given cell type. The authors of CIBERSORT recently released a newer version, CIBERSORTx (Newman et al. Nat. Biotech. 2019) which allows estimation of cell type-specific gene expression within a mixture. I believe a valuable extension to the analysis presented would be to compare estimated gene expression between COVID-19/IPF and healthy within particular subsets of interest, such as naïve/memory B cells. This could provide further clarity, for example by revealing whether the upregulation of integrin pathways is occurring within naïve B cells of COVID-19 patients or if it is occurring in other cell types or simply due to changes in B cell frequencies.**

**Response:**

We would like to thank Reviewer for his suggestion to re-analyze the results in Cibersort-X, but the data is too complex. For example, the specific genes screened by the software are currently unsupported by literature and are difficult to link to Naïve or memory B cells. Therefore, we used the LM22 immune cell gene signature from Cibersort to create specific signatures for Naïve or memory B cell, and then analyzed the bulk RNA-seq and single-cell RNA-seq to cross-validate the enrichment of the same signature in a wider variety of databases. Related gene expression is also specifically evaluated. We believe that such an approach would enhance the credibility of the results as well. All corrections and results will be described below.

**9. Figure 6: The results section describing changes in various interleukin signaling pathways is not clear about the disease specificity. It should mention that these changes are common to IPF and COVID-19. Furthermore, IL-4 signaling, which is upregulated in COVID-19 (and IPF), is known to induce proliferation and class switching in B cells (Gascan et al., J. Exp. Med. 1991). This appears at odds with the finding of increased naïve B cells in COVID-19 samples. How do the authors explain this apparent dichotomy? This should be addressed.**

**Response:**

Thanks to Reviewer's suggestion, we understand that it is difficult to directly relate the results of bulk RNA-seq samples to the effects of specific cell populations. However, it is possible that presenting the results of this kind of analysis may provide preliminary confirmation for other more in-depth studies, so we decided to present the results in supplementary data. On the other hand, we also tried to perform PID analysis in single cell RNA-seq, but the single B-cell diversity may be too divergent for simple dichotomy to obtain significant results, and thus the results are not presented. On the issue of IL-4 signaling, a report published this year indicated that the activation of IL-4 signaling may be related to the restore of DN2 B cell to naïve B cell [6]. Although this result is somewhat contrary to our additional inference, since the role of DN2 B cells in autoimmunity and the detailed activation mechanism remain to be elucidated, the up-regulation of IL-4 signal may also be a potential inhibitory mechanism indirectly induced by the over-activation of DN2 B cells, and preserving the relevant data may help future research progress.

Minor concerns:
**1. Figure 5B: What does the size of the circles represent?**
**Response:**

Thanks to reviewer's correction, the size of the circle, which represents the number of genes in the gene set, is clearly indicated in the recreated figure.
**2. Figure 6: Are these enrichments significant? The cutoffs used to determine inclusion in the figure should be stated in the legend**
**Response:**

Thanks to reviewer's correction, all enrichments have been filtered for FDR < 0.05 and are noted specifically in the recreated diagram and legends.
**3. Discussion: The authors refer to increased infiltration of different immune cells, it should be clarified that these are transcriptional estimates and not actual measurements of cell frequencies.**

**Response:**

Thanks to the reviewer for the corrections, which have been made to the text.

**GO:BP analysis based on GSEA reveals specific enrichment of B-cell-mediated innate humoral responses in the lungs of COVID-19 patients**

To understand the similarities and differences in the functional enrichment of gene sets in the lung tissues of COVID-19 and IPF patients compared to controls, the GO:BP (Gene Ontology Biological Processing) gene set was employed to evaluate a variety of biological responses, including immune responses.

Based on the GSEA results, Figure 5A lists the functional gene sets with NES scores greater than 2 (COVID-19: Red, IPF: Green; pre-screening criteria: nom. *P* < 0.05, FDR < 0.05). The purple text shows the set of genes that are co-enriched in COVID-19 or IPF patients, including Humoral immune response mediated by circulating immunoglobulin, B cell mediated immunity, complement activation, phagocytosis recognition, positive regulation of B cell activation and B cell receptor signalign pathway ranked first. Showing that an immune response caused by B-cell mediators may be a consistent phenomenon that causes damage to lung tissue in patients with COVID-19 or IPF. Green and red text represent the separately enriched gene sets in IPF or COVID-19 respectively. A number of gene sets related to lung fibrosis, including extracellular structure organization and collagen associated processes, were enriched in IPF. At the same time, a variety of genes related to B-cell proliferation, differentiation and maturation as well as adaptive immune response were also solely associated with IPF. Interestingly, none of these gene sets associated with B-cell maturation and differentiation were prominent in COVID-19, and instead were enriched for the innate immune response and FC receptor signaling pathway. This phenomenon is consistent with the results of the CIBERSORT-X analysis.

To understand which genes affect the CIBERSORT-X algorithm more, we ranked the top 100 genes enriched in naive B cell or memory B cell within LM22 signature document and performed Venn diagram analysis (Figure 5B). There are 20 genes that are not involved in intersection in both types of B cell, we define them separately as “Naïve B cell specific signature” and “Memory B cell specific signature”. In addition, to systematically visualize the enriched gene set clusters, we performed the enrichment map app in Cytoscape and set the enriched gene cluster in COVID-19 as red and IPF as green. The visualized results showed that clusters belong to immune cell activation were divergent, while the other clusters were clearly polarized. For example, the chromatin centromere and sister segregation gene sets are enriched only in COVID-19, while others are enriched only in IPF. We further overlap the defined B cell specific signature with the visualized clusters, the graphical representation showed that memory B cell specific signature was mostly associated with multiple immune-related gene sets of IPF. In contrast, the naive B cell specific signature was associated with COVID-19 enriched immune gene sets. These results are consistent with the data from the CIBERSORT-X analysis of B cell lineage (Figure 3). That is, in the lungs of COVID-19 patients, undifferentiated naïve B cells may be the main immune cells that elicit humoral immune response, which has been observed in clinical practice[1, 2].

**Figure 5 GO:BP functional enrichment map summary of GSEA in the lung tissues of COVID-19 and IPF patients.**
(A) The top NES-ranked enrichment results of biological processing by gene ontology (GO) functional enrichment analysis in IPF or COVID-19 (NES > 2; FDR < 0.05). The normalized enrichment score (NES) was presented in the X-axis, and the names of enriched functional gene sets were shown in the Y-axis. The color gradation on the right side indicates false discovery rate (FDR). Size represents the number of genes in the gene set. Color of the text represents whether the gene set was enriched in both (purple) or in individual type of disease (COVID-19: Red, IPF: Green). (B) Venn diagram: two circles represent the intersection status of the top 100 genes from the Naive B cell (Red) and Memory B cell (Green) subtypes extracted from the LM22 immune cell gene signatures, respectively. The detailed list of genes in the intersection is shown in the table below. Enrichment map: Commonality of positive correlations in the GO:BP gene sets after GSEA assessment using enrichment map visualization in COVID-19 (GSE150316, Red) and IPF (GSE124685 and GSE53845, Green). Single color: the node is positively correlated in only one disease condition; Mixed: the node is positively correlated in two or three databases. Diamond: Naïve or Memory B cell specific signature, genes in the signature were shown in the table below. Lines connecting nodes or diamonds represent the degree of overlapping between two gene sets. Criteria for significance screening: *P*-value <0.05, FDR <0.05.

**Differences in B-cell clustering subtypes between COVID-19 and IPF patients verified in single-cell RNA sequencing databases**

CIBERSORT and similar deconvolution methods can be used to analyze the distribution of immune cells in the Bulk RNA-seq data, but the analysis of the signal pathways may not be accurate due to the overlap of activation between different cells. In order to further validate the phenomenon observed in bulk RNA-seq, we searched several publicly available single-cell RNA-sequencing databases and restricted the selecting criteria to those sampling from lung-associated body fluids or tissues. Chua et al. published single-cell RNA sequencing analysis of respiratory fluid samples from 20 patients with COVID-19 mild to severe disease, and Liao et al. published single-cell RNA sequencing analysis of bronchoalveolar lavage fluid (BALF) from 9 patients with COVID-19 mild to severe disease (GSE145926). On the other hand, Morse et al. published single-cell RNA sequencing analysis of lung tissues from IPF patients (GSE128033). According to the annotation provided by the authors, we circled B-cell populations from patients or healthy donors and evaluated the overall percentages of expression and the average standardized expression of each gene in the B-cell specific signatures. The average standardized expression of all genes was further analyzed (Figure 6). The results showed that genes in naive B cell specific signature was significantly higher in COVID-19 patient B cells, whereas genes in memory B cell specific signature were generally increased in IPF patient B cells, echoing our observations in bulk RNA-seq databases.

**Figure 6 Validation of the phenotypes of B-cell populations in single-cell RNA sequencing of lung-associated body fluid samples from COVID-19 and IPF patients.**
UMAP shows the visualized distribution of whole cells in single-cell RNA sequencing data from two COVID-19 databases (A) and IPF database (B). the “B-cell clusters” annotated by the original authors are shown in red, the unassigned cells are shown in gray. B-cell clusters are further colored red and blue to represent those from disease or healthy donors (HC) respectively. The proportion and average expression of genes related to Naive or Memory B cells in the B-cell population are shown as dot plots. Relative gene expression is shown in color and the overall B-cell expression gene percentage is shown in dot size (%). The mean Z scores of all genes’ expression in B cell populations from HC or disease groups are shown in Violin plot. Statistical significance is analyzed using Welch’s t-test approach. *: *P* < 0.05, **: *P* < 0.01, ***: *P* < 0.001. The actual *P*-values are shown under the asterisks as well.

**B cells from patients with severe COVID-19 have genetic phenotype similar to antibody-secreting cells, and the percentage of B cell infiltration appears to correlate with the severity of the disease.**

Given the results of the bulk RNA seq analysis, we were curious whether any specific distinctions existed between the B-cell lineage populations of patients with mild or severe COVID-19. Firstly, we divided the B cells from COVID-19 in two single-cell databases by disease severity and analyzed them by GSEA together with gene sets from C7 immune signature that belonged to B cell lineage, and then we cross match the two databases with the gene set enriched in moderate disease. The results showed that B cells from COVID-19 patients with severe symptoms had a tendency to be antibody secreting cells, such as plasma cells, plasmablast, IgM memory B cells, Follicular B cells, and unstimulated B cells compared to those from patients with mild symptoms (Figure 7A). Taken together with the aforementioned observations, we hypothesized that B cells from the lungs of COVID-19 critically ill patients may be associated with DN2 B cells, which have been considered in recent years to be the main inducer of extrafollicular response [3, 4]. In-depth analysis of DN2 B-cell related genes revealed that the overall expression ratio and average expression of genes that were thought to be reduced in DN2 B cells, such as CD21, CXCR5, IRF8 and TRAF5, were lower in B cells from critically ill patients, especially in samples from BALF (Figure 7B). Further, we analyzed the ratio of B-cell infiltration and the clinical characteristics of the patients and showed that when B-cell infiltration > 1%, there was a longer hospitalization and ICU duration, a higher proportion of severe disease (66.7%), a higher proportion of no recovery (33.3%) and a higher proportion of multi-organ failure (55.6%), indicating an association between increased B-cell infiltration and severity of disease in COVID-19 patients (Figure 7C).

**Figure 7 Assessing the phenotypic and clinical status differences of B-cell populations between mild and severe symptoms of COVIF-19.**

(A) Schematic diagram of the GSEA analyzing process for B cells of mild and severe COVID-19 patients in different databases. GSEA results were cross-referenced and the overlapping enriched data sets of the two databases were listed. The NES scores of each gene set are additionally represented by shades of red to represent the relative enrichment level. Those considered relevant to the B-cell population of patients with severe COVID-19 in the two immune cell comparisons are marked in red. (B) The dot plots represent the overall expression ratio and average expression of genes thought to be related to antibody-secreting cells and DN2 B cells. The overall expression percentage is represented by the size of the dots and the average expression is represented by the color. (C) Comparison of B-cell infiltration ratio and clinical status of patients with COVID-19. Box plots are shown for patients with B cell infiltration ratio < 1% (Black) or > 1% (Red) for duration of hospitalization and ICU. Pie charts show the severity, recovery and complications of multi-organ failure in patients with different levels of B-cell infiltration.

1.	Ni, L., et al., *Detection of SARS-CoV-2-Specific Humoral and Cellular Immunity in COVID-19 Convalescent Individuals.* Immunity, 2020.

2.	Long, Q.X., et al., *Antibody responses to SARS-CoV-2 in patients with COVID-19.* Nat Med, 2020.

3.	Jenks, S.A., et al., *Extrafollicular responses in humans and SLE.* Immunol Rev, 2019. **288**(1): p. 136-148.

4.	Jenks, S.A., et al., *Distinct Effector B Cells Induced by Unregulated Toll-like Receptor 7 Contribute to Pathogenic Responses in Systemic Lupus Erythematosus.* Immunity, 2018. **49**(4): p. 725-739 e6.

5.	Cavalli, E., et al., *Transcriptomic analysis of COVID19 lungs and bronchoalveolar lavage fluid samples reveals predominant B cell activation responses to infection.* Int J Mol Med, 2020. **46**(4): p. 1266-1273.

6.	Hsu, H.-C., et al., *IL-4 synergizes with low-dose IL-2 to restore systemic lupus erythematosus B cells at the resting naive status.* The Journal of Immunology, 2020. **204**(1 Supplement): p. 218.8-218.8.
