# Supplementary material for: In silico immune infiltration profiling combined with functional enrichment analysis reveals a potential role for naïve B cells as a trigger for severe immune responses in the lungs of COVID-19 patients
Source: PLoS One. 2020 Dec 2;15(12):e0242900. doi: 10.1371/journal.pone.0242900 (PMC7710067; doi:10.1371/journal.pone.0242900)
Supplement: Attachment — Submitted filename: Rebuttal letter 2nd.docx [file pone.0242900.s003.docx]

**Reviewer #2: Summary:**

**The authors have addressed most of my initial concerns. However, the updated analysis and results suggest that naïve B cells are not elevated in the BALF of severe COVID-19 patients relative to moderate cases, but that the B cells of severe patients may be enriched in antibody-secreting cells instead. This does not implicate naïve B cells in severe immune responses to COVID-19 and the conclusions of the manuscript need to be altered to be in agreement with these new findings.**

**Major concerns:**

**1. Figure 5B: How do the authors define ‘top gene’ in the memory v naïve comparison? Is this based on the top 100 most highly expressed genes from the LM22 signature for both the memory and naïve B cell subsets? This is not an ideal way to define a naïve vs memory specific signature, because it is not guaranteed to identify the most distinctive genes. The 20 genes unique to naïve could be rank 101-120 in memory, just missing the cutoff, for example. Therefore, the authors should validate their analyses by using an alternative memory vs naïve signature and see if they produce consistent results. One approach could be to use up/down DEG memory vs naïve B cell gene sets included in the C7:immunologic signature gene sets from MSigDB.**

**Response:**

We really appreciate the suggestions, and we have indeed tried and considered a number of methods similar to this. We initially spent a lot of time searching the literature for a more representative gene-set of memory and naïve B-cell, but most of the literature only had one or two genes (e.g. CD27 for memory B cell) as representative of the B-cell population. Considering that we initially observed this phenomenon from the results of the Cibersort analysis and aimed to narrow down the results to focus on differences in expression of immune cell related genes in further single-cell sequencing databases, we finally decided to use the current method for the evaluation. There are two reasons for choosing the top 100 genes: firstly, in the LM22 document, the weighting (numbers of each gene in the LM22 document ) of the top 10 immune cell-specific genes is over 10,000 and drops to a few hundred after the top 100, and the weighting of the genes further down the list is insignificant for the software to determine the immune infiltration score of the immune cells. Secondly, among the immune cell-specific genes generated by this method, although there are indeed problems as you mentioned, most of them have more than twofold differences in the weight of genes in the LM22 naive and memory cell gene set, so we think that they still efficient to discriminate between the two types of B cells. Further, a number of genes are listed as naive or memory B-cell-specific biomarkers in the single-cell biomarker database (PanglaoDB, https://www.panglaodb.se/index.html) (Naive: ZNF286A, MEP1A, FCER2, UGT1A8, BEND5, BCL7A, P2RY14; Memory: TNFRSF13B, CD27, CD86, IL7, AIM2) , and most of the biomarker genes fall in the 50th to 100th rank, showing that this method is capable of accurately reflecting biomarker genes in specific immune cell type. In addition, we also used the same method to find specific genes representing memory and naive B cells in the MSigDB C7:Immunology signature gene set (GSE13411), with even fewer genes intersecting with those from single-cell biomarker database, and thus finally decided not to adopt it for analysis.

2. Figure 7: The moderate versus severe B cell comparison here is interesting and informative. However, as the authors correctly point out, the results indicate that severe patients likely have increased plasma cells or other antibody-secreting cells relative to mild/moderate patients. These cells, as well as DN2 B cells, are not naïve. Therefore, this would suggest that naïve B cells are not involved in the pathology of severe COVID-19, but that potentially antibody-secreting cells are instead. This is at odds with the conclusions of the manuscript, including the title. Unless the authors have some alternative explanation as to why these results do not implicate antibody-secreting cells rather than naïve B cells in severe COVID-19, the conclusions of the manuscript need to be changed to better match these findings.

Response：Thank you for your approval. With regard to the association of antibody-secreting cells with naive cells, we did not adequately describe the context in the current manuscript and have therefore corrected it as shown below:

“As possessing both naïve B-cell and antibody-secreting cell properties, these cells are similar to the DN2 B-cells found in systemic lupus erythematosus proposed by Tipton et al. The researchers found that they are derived from naïve B cells and appear to be precursors of plasma cells [1]. The term double negative (DN) comes from the absence of immunoglobulin D and memory B-cell marker CD27 [2]. DN B cells are further classified into DN1(CXCR5^+^) and DN2(CXCR5^-^) by whether CXCR5 is expressed or not [3]. An increase in IRF4 and a decrease in IRF8 are thought to be associated with the promote naïve B cell differentiation into DN2 [4, 5]. The process of induced DN2 B-cell differentiation is accompanied by a decrease in TRAF5 and CD21 and an increase in CD11c [6]. These DN2 B cells are usually found outsides of B-cell follicles rather than germinal centers and may therefore be genetically phenotypically different from GC-derived memory B cells.”

Regarding the difference between antibody-secreting cells (ASC) and naive cells, it is true that it is difficult to connect the two in previous definitions. However, recent studies investigating DN2 B cells that induce Systemic Lupus Erythematosus (SLE) indicate that these B cells are directly stimulated by follicular helper T cells and differentiate into plasma-like cells that secrete large amounts of antibodies while retaining properties of naïve cells to some extent [6]. These implications led us to try to assess whether the infiltrating B-cells in patients with severe COVID-19 have a tendency to be DN2 B-cells.

We understand that there is insufficient evidence to directly define these ASC-like naïve cells as DN2 B cells by inference for the further conclusion in this article, because there is no credible gene set or experiment to define these naïve B cells as DN2 B cells so far. Therefore, we decided to keep the initial “naive B-cells” in the title, which showed a similar tendency in both bulk RNA-seq and single cell sequencing results and included the postulated characteristics of DN2 B cell in the single cell sequencing analysis and discussion as a clue for further research.

**Minor concerns:**

**1. The end of the introduction mentions ‘anti-secretory cells’, is this supposed to be antibody-secreting cells (as in the abstract)?**

**Response：**

Thank you for correcting this typo, which we have corrected as follows:

“Further analysis of the defined B-cell population using single-cell RNA sequencing databases showed that the B-cells from COVID-19 patients not only tended to be naïve B-cells, but also tended to be antibody-secreting cells in patients with severe disease, and the proportion of B-cell infiltration seemed to correlate with the severity of the disease.”

**2. Figure 5A: This panel might be easier to interpret if these results were separated into 3 plots (COVID-19, IPF, and common)**

**Response：**

Thank you for your suggestion, we have recreated the following figure:

**3. Figure 6: The Liao and Morse datasets both have ~40% B cells in control samples, but the Chua dataset has < 1% B cells in control samples. Why do these controls have such a low frequency of B cells? Are they appropriate controls?**

**Response：**

With regard to the differences in B-cell infiltration in healthy donors, we suggest that this may be due to sampling methods. In the Liao and Morse datasets, samples were collected from patients' bronchoalveolar lavage fluid, whereas in Chua dataset, most samples were collected from respiratory mucosa by swab only. Therefore, in the Chua dataset, samples from healthy donors may have significantly different B-cell population due to lower mucosal secretions. We understand that this is an experimental limitation and that extreme differences in quantity may also cause bias in the results. However, this database is an important source of evidence for the extent of disease and immune cell infiltration in patients, and we felt it was necessary to perform consistent analyses across all databases, and therefore used these small numbers of B cells as controls.

**4. Figure 7B: What is the source for these DN2 B cell gene markers? Are these differences significant?**

**Response:**

Thank you for the consideration, these markers are based on the gene markers used in several widely cited papers discussing DN2 B-cells, so we trust that these markers are approved. The relevant genes and literature have been added to the context as follows:

“As possessing both naïve B-cell and antibody-secreting cell properties, these cells are similar to the DN2 (Double negative) B-cells found in systemic lupus erythematosus proposed by Tipton et al. The researchers found that they are derived from naïve B cells and appear to be precursors of plasma cells [1]. The term double negative (DN) comes from the absence of immunoglobulin D and memory B-cell marker CD27 [2]. DN B cells are further classified into DN1(CXCR5^+^) and DN2(CXCR5^-^) by whether CXCR5 is expressed or not [3]. An increase in IRF4 and a decrease in IRF8 are thought to be associated with the promote naïve B cell differentiation into DN2 [4, 5]. The process of induced DN2 B-cell differentiation is accompanied by a decrease in TRAF5 and CD21 and an increase in CD11c [6]. These DN2 B cells are usually found outsides of B-cell follicles rather than germinal centers and may therefore be genetically phenotypically different from GC-derived B cells.”

The relevant statistical difference calculations have been supplemented as shown in the figure below:

1.	Tipton, C.M., et al., *Diversity, cellular origin and autoreactivity of antibody-secreting cell population expansions in acute systemic lupus erythematosus.* Nat Immunol, 2015. **16**(7): p. 755-65.

2.	Wei, C., et al., *A new population of cells lacking expression of CD27 represents a notable component of the B cell memory compartment in systemic lupus erythematosus.* J Immunol, 2007. **178**(10): p. 6624-33.

3.	Ehrhardt, G.R., et al., *Discriminating gene expression profiles of memory B cell subpopulations.* J Exp Med, 2008. **205**(8): p. 1807-17.

4.	Nutt, S.L., et al., *The generation of antibody-secreting plasma cells.* Nat Rev Immunol, 2015. **15**(3): p. 160-71.

5.	Xu, H., et al., *Regulation of bifurcating B cell trajectories by mutual antagonism between transcription factors IRF4 and IRF8.* Nat Immunol, 2015. **16**(12): p. 1274-81.

6.	Jenks, S.A., et al., *Distinct Effector B Cells Induced by Unregulated Toll-like Receptor 7 Contribute to Pathogenic Responses in Systemic Lupus Erythematosus.* Immunity, 2018. **49**(4): p. 725-739 e6.
